# Phyletic Distribution and Diversification of the Phage Shock Protein Stress Response System in Bacteria and Archaea

**DOI:** 10.1128/msystems.01348-21

**Published:** 2022-05-23

**Authors:** Philipp F. Popp, Vadim M. Gumerov, Ekaterina P. Andrianova, Lisa Bewersdorf, Thorsten Mascher, Igor B. Zhulin, Diana Wolf

**Affiliations:** a Institute of Microbiology, Technische Universität Dresden, Dresden, Germany; b Department of Microbiology, The Ohio State University, Columbus, Ohio, USA; c Medical Clinic III, Gastroenterology, Metabolic Diseases and Intensive Care, University Hospital RWTH Aachen, Aachen, Germany; d Translational Data Analytics Institute, The Ohio State University, Columbus, Ohio, USA; University of Waterloo

**Keywords:** PspA, PspC, comparative genomics, signal transduction, cell envelope stress response, *Bacillus subtilis*, *Escherichia coli*, Psp response

## Abstract

Maintaining cell envelope integrity is of vital importance for all microorganisms. Not surprisingly, evolution has shaped conserved protein protection networks that connect stress perception, transmembrane signal transduction, and mediation of cellular responses upon cell envelope stress. The phage shock protein (Psp) stress response is one such conserved protection network. Most knowledge about the Psp response derives from studies in the Gram-negative model bacterium Escherichia coli, where the Psp system consists of several well-defined protein components. Homologous systems were identified in representatives of the *Proteobacteria*, *Actinobacteria*, and *Firmicutes*. However, the Psp system distribution in the microbial world remains largely unknown. By carrying out a large-scale, unbiased comparative genomics analysis, we found components of the Psp system in many bacterial and archaeal phyla and describe that the predicted Psp systems deviate dramatically from the known prototypes. The core proteins PspA and PspC have been integrated into various (often phylum-specifically) conserved protein networks during evolution. Based on protein domain-based and gene neighborhood analyses of *pspA* and *pspC* homologs, we built a natural classification system for Psp networks in bacteria and archaea. We validate our approach by performing a comprehensive *in vivo* protein interaction study of Psp domains identified in the Gram-positive model organism Bacillus subtilis and found a strong interconnected protein network. Our study highlights the diversity of Psp domain organizations and potentially diverse functions across the plethora of the microbial landscape, thus laying the ground for studies beyond known Psp functions in underrepresented organisms.

**IMPORTANCE** The PspA protein domain is found in all domains of life, highlighting its central role in Psp networks. To date, all insights into the core functions of Psp responses derive mainly from protein network blueprints representing only three bacterial phyla. Despite large overlaps in function and regulation, the evolutionary diversity of Psp networks remains largely elusive. Here, we present an unbiased protein domain- and genomic context-centered approach that describes and classifies Psp systems. Our results suggest so-far-unknown Psp-associated roles with other protein networks giving rise to new functions. We demonstrate the applicability of our approach by dissecting the Psp protein network present in Bacillus subtilis and demonstrate Psp domains working in concert with other cell envelope stress response systems. We find that the Psp-like protein universe reflects a surprising diversity within the bacterial and archaeal microbial world.

## INTRODUCTION

The cell envelope is an essential, multilayered, and complex structure, which physically separates bacterial cells from the environment. In their structural composition, Gram-positive and Gram-negative bacteria both share the cytoplasmic membrane and the cell wall. While the latter is much thicker in Gram-positive bacteria, the Gram-negative envelope additionally harbors an outer membrane (1). The cytoplasmic membrane is the functional barrier of the cell and fulfills crucial tasks, such as serving as a diffusion barrier, allowing the generation of the proton motive force (PMF), and providing a platform for protein-protein interaction (1, 2). Due to its essentiality, it is indispensable for prokaryotes to closely monitor and maintain their cell envelope integrity (3). This involves stimulus perception and signal transduction modules that comprise complex regulatory networks orchestrating a cell envelope stress response (CESR), which is activated when a cell is challenged with adverse conditions such as envelope-perturbating antimicrobial compounds (4, 5).

**FIG 1:**
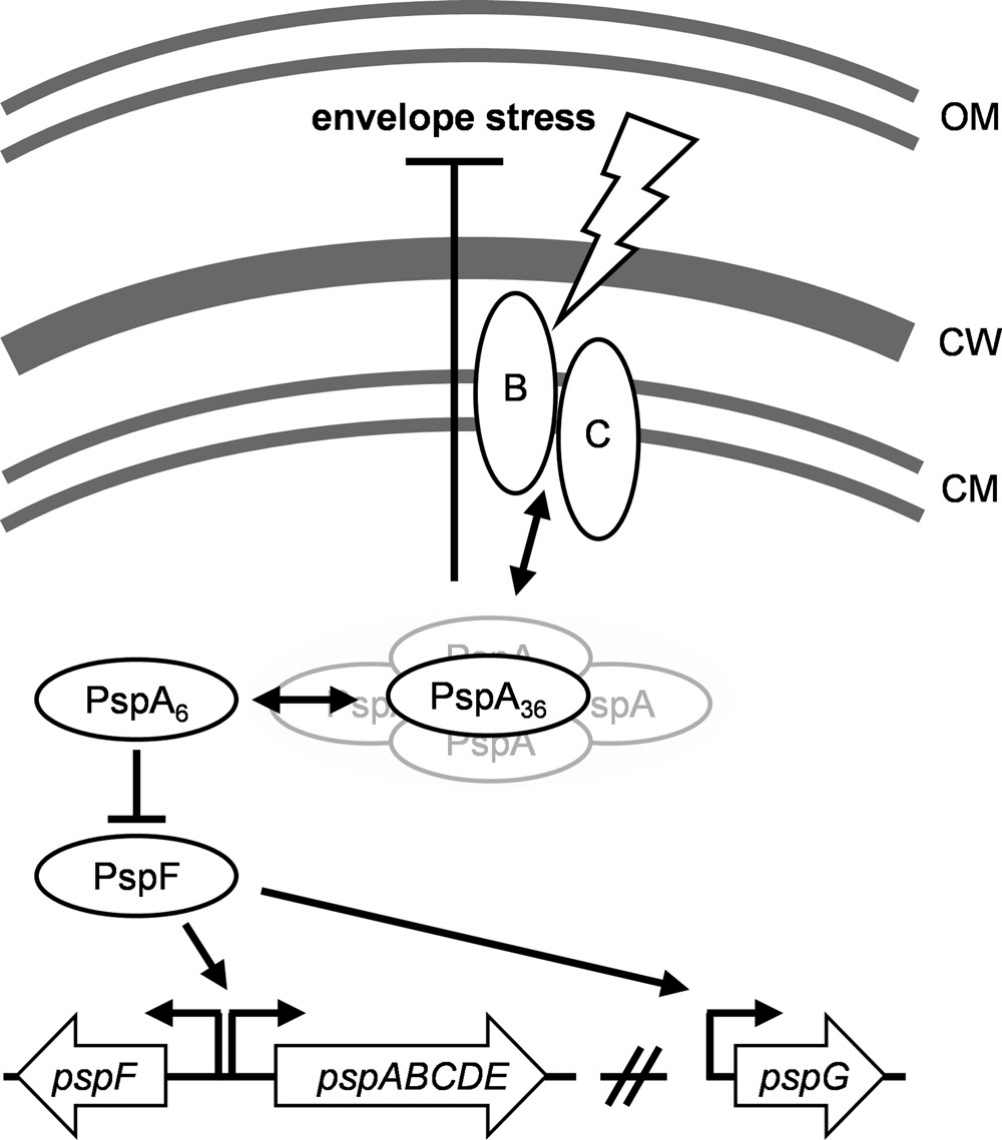
The phage shock protein response in E. coli. Upon stimulus perception mediated by the signal detectors PspB and PspC, PspA oligomerizes and presumably supports membrane integrity at the site of damage perception. The transition of the PspA polymerization state (either mediated by or independent of PspB/C) causes the release of the transcriptional regulator PspF, enabling the transcription of the *pspA–E* operon. In E. coli, the orphan *pspG* gene is the only other known target of PspF; however, its biological function within the Psp response is still unknown. OM, outer membrane; CW, cell wall; CM, cytoplasmic membrane.

One such system, the phage shock protein (Psp) response, has been studied in bacteria, and one component of this system, PspA, has been identified in archaea and plants (6). Initial studies in Escherichia coli revealed the strong induction of PspA protein expression during phage infection accompanied by the production of the phage protein pIV, reassembling an outer membrane pore-forming secretin (7). Subsequent studies on the Psp network identified various inducers, including other secretins, elevated temperature or osmolarity, or interference with fatty acid biosynthesis (8–10). In E. coli, PspA is encoded in the *pspABCDE* operon, and expression levels are regulated by the PspF activating protein via σ^54^ (Fig. 1) (7, 11). Under noninduced conditions, PspA forms a complex with PspF, thus silencing its own transcription (Fig. 1) (12). Dependent on the stimulus perceived, PspB and PspC function as signaling units and initiate the disassembly of the PspA-PspF complex, thereby enabling the activation of the system (13–15). As a consequence, PspA proteins form a 36-meric donut-shaped oligomer that supports membrane integrity at the site of damage perception (Fig. 1) (16). Interestingly, the activation of the Psp system in E. coli by heat was shown to be PspB and PspC independent and required solely PspA (13). Thus, PspA functions as (i) a regulator, (ii) a sensing unit, and (iii) an effector protein, substantiating its key role within the Psp network. The biological function and physiological significance of the remaining Psps, PspD and PspE, are still unclear (15, 17). PspF also regulates the orphan *pspG* gene, which is the only other known PspF target in E. coli. However, the role of PspG in the Psp response is not fully understood (15, 18). It has been shown that PspG is involved in the signaling cascade upon the filamentous phage secretin protein IV stress response in E. coli and Salmonella enterica serovar Typhimurium LT2 strains (19, 20). Deletions in the *psp* operon of E. coli show mild phenotypes, such as growth defects in late stationary phase (15, 21). However, mutagenesis of *psp* genes on top of known stress response genes showed increased sensitivity to antimicrobial compounds (22). In the light of some multidrug effector proteins relying on the PMF to drive the respective resistance pumps, the overexpression of PspA can represent a compensatory role. This is further supported by observations of E. coli persister cells that are known to develop multidrug tolerance depicting elevated *psp* expression (23, 24).

In contrast, the Psp system in Yersinia enterocolitica is of importance for bacterial survival when the virulent Ysc type III secretion system is expressed during host infection (25). It has been shown that the deletion of *pspC* results in reduced virulence and growth defects (25, 26). Detailed research on the Psp response also revealed the roles of the membrane proteins PspB and PspC as dually (positive and negative) acting regulators in *psp* operon expression in Y. enterocolitica (27). Surprisingly, PspA plays no essential role in terms of cell growth and bacterial survival during host infection, whereas PspBC are needed to protect the cells from secretin-induced death (28, 29). Furthermore, the genetic organization of the Psp locus in Y. enterocolitica differs from that in E. coli, PspC contains an N-terminal extension that is involved in *psp* gene expression regulation and is not present in E. coli, and Y. enterocolitica *psp* operons are accompanied by *ycjXF* genes encoding both non-Psp-response-associated domain of unknown function (DUF)-containing proteins. Moreover, a PspE homolog is missing in Y. enterocolitica (15, 25).

In the Gram-positive model organism Bacillus subtilis, the PspA homolog termed LiaH also forms oligomeric ring structures during cell envelope stress (CES), thus substantiating the role of PspA-like proteins in supporting membrane integrity (30, 31). In contrast to the regulation in E. coli, B. subtilis *liaH* is controlled by the two-component system (TCS) LiaRS, which strongly induces the expression of the *liaIH* operon upon perceiving CES. In addition to LiaH, this operon encodes a membrane anchor protein, LiaI, that facilitates LiaH recruitment to the cytoplasmic membrane (5, 30). B. subtilis also encodes a second PspA protein and a PspC domain-containing protein in separate operons. The domain diversity of the Psp system in Gram-positive bacteria was also described in a recent study of the actinobacterium Corynebacterium glutamicum, where the PspC domain was found as the N-terminal input module of a histidine kinase, embedded in a three-component system responsive to CES (32).

Previous genomics studies focused on analyzing the Psp system in only three bacterial phyla, *Proteobacteria*, *Firmicutes*, and *Actinobacteria*, with experimentally studied representatives (33, 34), whereas the newest genome-based taxonomy defines more than 100 bacterial and archaeal phyla (35). Thus, our knowledge of the diversity and distribution of the Psp system throughout prokaryotes is very limited. To fill this gap, we performed a large-scale genomic analysis of Psp-like networks in bacteria and archaea by analyzing more than 22,000 genomes, representing all bacterial and archaeal phyla for which sufficient genomic data are available (35). First, we analyzed the distribution of Psp-specific domains throughout different phylogenetic ranks. We then performed in-depth profiling of putative Psp networks encoded in each genome in the data set. The PspA and PspC domains showed the highest diversity with respect to phyletic distribution and associations with other domains within a single protein. By analyzing the domain architectures and genetic neighborhoods of PspA and PspC, we identified new genomic organizations and provided context-specific knowledge enabling predictions of novel Psp network architectures and domain combinations. Using a broad bacterial two-hybrid (B2H) screen, we confirmed that multiple Psp domains present in an organism can work in concert, forming the novel concept of a Psp-like CES network. We thus validate our *in silico* analysis and experimentally dissect the Psp network of B. subtilis, which consists of 14 proteins, some of which contain known and predicted Psp domains that are encoded in three separate genomic locations.

## RESULTS AND DISCUSSION

### Genomic perspective on the phage shock proteins.

**FIG 2:**
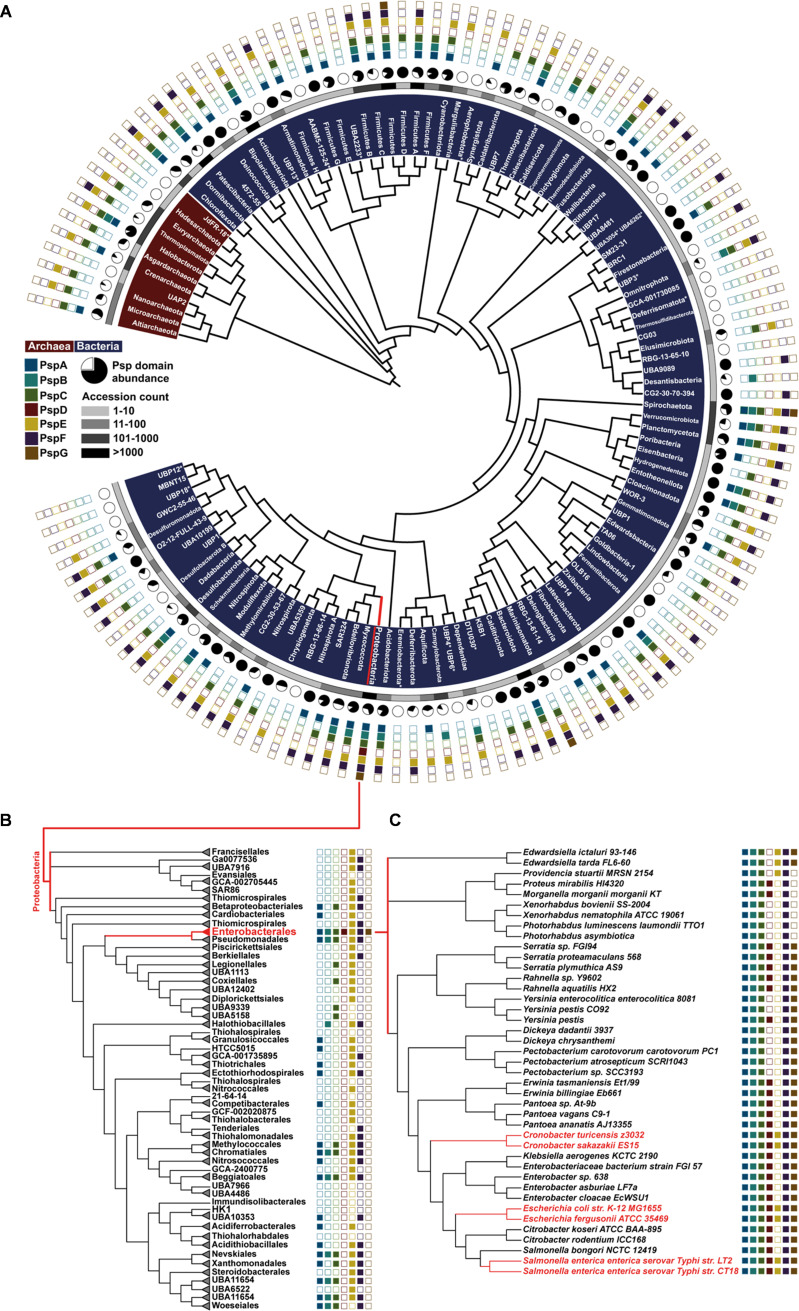
Phylogenetic diversification of Psps in bacteria and archaea. (A) Phylogenetic representation of bacterial and archaeal phyla (phylogenetic tree adapted from AnnoTree [80]). The inner circle represents a scale indicating the number of analyzed genomes per phylum. Black/white circles highlight the abundance of genomes harboring any Psp. Most outer squares show Psp domains found across all genomes of the respective phyla. For a comprehensive data set, see Data_S1 at OpARA (http://dx.doi.org/10.25532/OPARA-117). (B) Phylogenetic tree applying the neighbor-joining algorithm based on concatenated proteins (35) of randomly selected representatives within the order *Gammaproteobacteria*. Genomes were screened for Psp domain presence (see Materials and Methods; also see Data_S2 at OpARA [http://dx.doi.org/10.25532/OPARA-117]). (C) Phylogenetic tree using the maximum likelihood algorithm based on concatenated proteins (35) of genomes within the *Enterobacteriaceae* family. Representatives were probed for the presence of Psp domains (see Data_S2 at OpARA [http://dx.doi.org/10.25532/OPARA-117]).

The genomic sequence space of the microbial world is rapidly increasing, with close to 150,000 bacterial and more than 2,000 archaeal genomes currently classified in the Genomic Taxonomy Database (GTDB) (v86) (35). But the sheer size of this data set does not necessarily reflect the phylogenetic diversity in nature, as the number of sequenced bacterial genomes is currently heavily biased toward three bacterial phyla, *Proteobacteria*, *Firmicutes*, and *Actinobacteriota*, comprising more than two-thirds of the available genomic data (35). This leaves the remaining bacterial phyla highly underrepresented and demands an unbiased approach to tackle genomic data. We therefore first generated such a data set, containing approximately 22,000 genomes that represent 99 bacterial and 10 archaeal phyla (Fig. 2A; also see Materials and Methods). This set of genomes is a balanced data set compiled and used for classification by the GTDB (35). Next, we applied the hidden Markov models (HMMs) of each phage shock protein (Psp) domain to all genomes to screen our data set for the diversity of the Psp systems throughout bacteria and archaea. Next, we analyzed the phylogenetic distribution of each domain from the known proteobacterial Psp network (Fig. 2A). Eighty-three bacterial and 7 archaeal phyla contain individual genomes encoding Psp domains, but the abundance of Psp-positive genomes within these phyla varied substantially (Fig. 2A, black/white circles). About 45% and 60% of the phyla contained genomes encoding the effector protein PspA and the signaling protein PspC, respectively. In the context of Psp domains, this wide phylogenetic distribution highlights that the PspA and PspC domains represent the core of a Psp network architecture (Fig. 1A) (36). This was supported by the rapidly descending number of phyla encoding any other domain of the Psp system, such as the signaling protein PspB, which was found in only 26% of the analyzed phyla.

The transcriptional activator PspF is a special case: while it was present in 50% of the phyla, its multiple-domain composition may not be restricted to the Psp response but might instead be associated with a variety of additional cellular processes (37). Since the PspF regulator of the Psp response has so far been described only in *Enterobacteriaceae*, the occurrence of orthologous proteins, containing a PspF domain, outside this phylogenetic group is difficult to strictly associate with the Psp response since PspF is composed of multiple protein domains (15, 38). The same accounts for the single-domain protein PspE, a rhodanese that catalyzes sulfur transfer from thiosulfate to thiophilic acceptors in a variety of cellular processes beyond the involvement in Psp networks (39, 40). PspD and PspG, the two remaining domains present as Psp members in the classical system of E. coli, have the narrowest phylogenetic distribution. The PspG domain was found in only four genomes from three phyla outside *Proteobacteria*, while the PspD domain was restricted to *Proteobacteria*. Since only proteobacterial genomes harbor all Psp domains, we next focused on the Psp distribution within this phylum. First, a representative collection of 7,500 proteobacterial genomes was resolved on the taxonomic level of order (Fig. 2B; see also Materials and Methods). Here, the complete set of Psp members was found only within *Enterobacterales*, whereas the remaining orders showed only partial representations of the Psp system (Fig. 2B). Within the order *Enterobacterales*, we next resolved the Psp domain distribution at the taxonomic rank of family (Fig. 2C). Remarkably, the complete Psp system, as found in E. coli, was present in only the closest relative species, such as Salmonella enterica. More distantly related species of the same family, such as Photorhabdus luminescens, harbor Psp domains comprising only the core functions of PspABC. The lack of single or multiple Psp domains within close relatives of E. coli could suggest slimmed-down network architectures or the existence of alternative blueprints of the Psp system even within *Enterobacterales* (Fig. 2C) (15).

### PspA and PspC domains are the most prevalent Psp network members.

**FIG 3:**
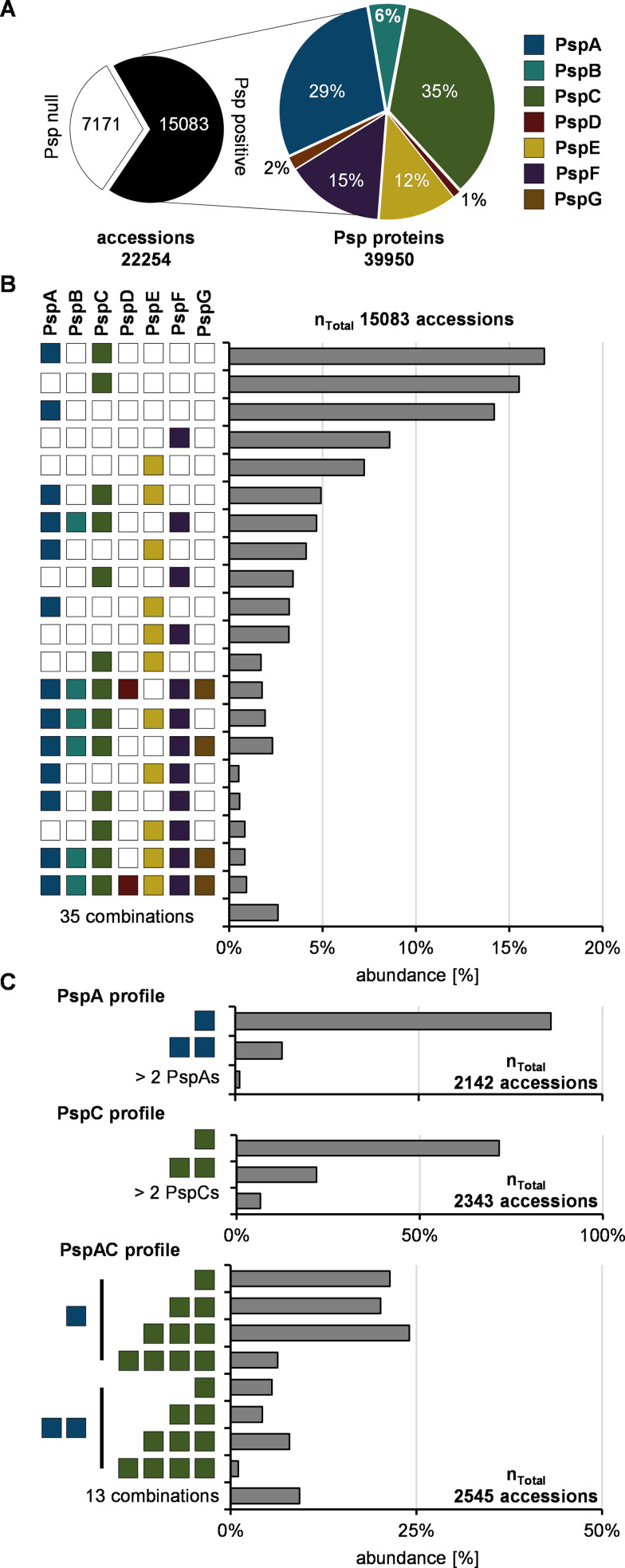
Psp profiles resolved on the genomic level. (A) Screening of the full data set for the presence/absence of Psp domains and their relative abundance (see Data_S1 at OpARA [http://dx.doi.org/10.25532/OPARA-117]). (B) Descending categories of genomes according to the Psp domain profiles found. Abundance was set relative to Psp-positive genomes in panel A (see Data_S3 at OpARA [http://dx.doi.org/10.25532/OPARA-117]). (C) In-depth analysis of PspA- or -C- and PspAC-containing genomes probed for the abundance of multiple proteins per genome containing further PspA or -C domains (see Data_S3 at OpARA [http://dx.doi.org/10.25532/OPARA-117]).

The indicated diversity of the Psp domain occurrences prompted us to next identify the Psp profiles of each genome within the data set to establish a comprehensive overview of Psp domain patterns and to resolve the conservation of Psp network organization in bacteria and archaea. Toward this goal, we analyzed the cooccurrence of Psp members in each genome. We first screened the data set for the presence or absence of individual Psp domains. Sixty-eight percent of the approximately 22,000 genomes encoded at least one Psp domain-containing protein, while close to 7,200 genomes lacked any Psp domain (Fig. 3A; see also Data_S1 at OpARA [http://dx.doi.org/10.25532/OPARA-117]). More than 60% of the 40,000 Psp proteins identified contained either PspA or PspC domains (Fig. 3A), including genomes containing multiple copies of the same Psp domain. For example, we identified eight PspA domain proteins in the genome of Aneurinibacillus tyrosinisolvens, which was recently isolated from methane-rich seafloor sediments (41) (see Data_S1 at OpARA [http://dx.doi.org/10.25532/OPARA-117]).

We next categorized the Psp domain distribution and abundance by assigning a Psp profile to each genome. From approximately 15,000 genomes encoding any Psp domain, more than 45% harbored only PspA, PspC, or PspA and PspC domain proteins (Fig. 3B; see also Data_S3 at OpARA [http://dx.doi.org/10.25532/OPARA-117]). In comparison, only about 1% (79 genomes from *Enterobacteriaceae*) encoded the full repertoire of the Psp domain network (Fig. 3B). These observations strongly support the hypothesis that most Psp networks can deviate in their architecture from the proteobacterial blueprint exemplified by E. coli or S. enterica. More than 10% of the genomes containing only PspA or PspC encoded their multiple paralogs (Fig. 3C; see also Data_S3 at OpARA [http://dx.doi.org/10.25532/OPARA-117]). These duplications were found in 16 phyla for PspA and 23 for PspC. In genomes harboring both PspA and PspC, many encoded multiple PspC domains. This may suggest that either the signaling properties of PspC domains serve beyond their relationship linked to PspA or different stimuli are integrated via individual PspC proteins to enhance Psp response specificity (Fig. 3C).

### Domain combinatorics and diversity of PspA and PspC.

**FIG 4:**
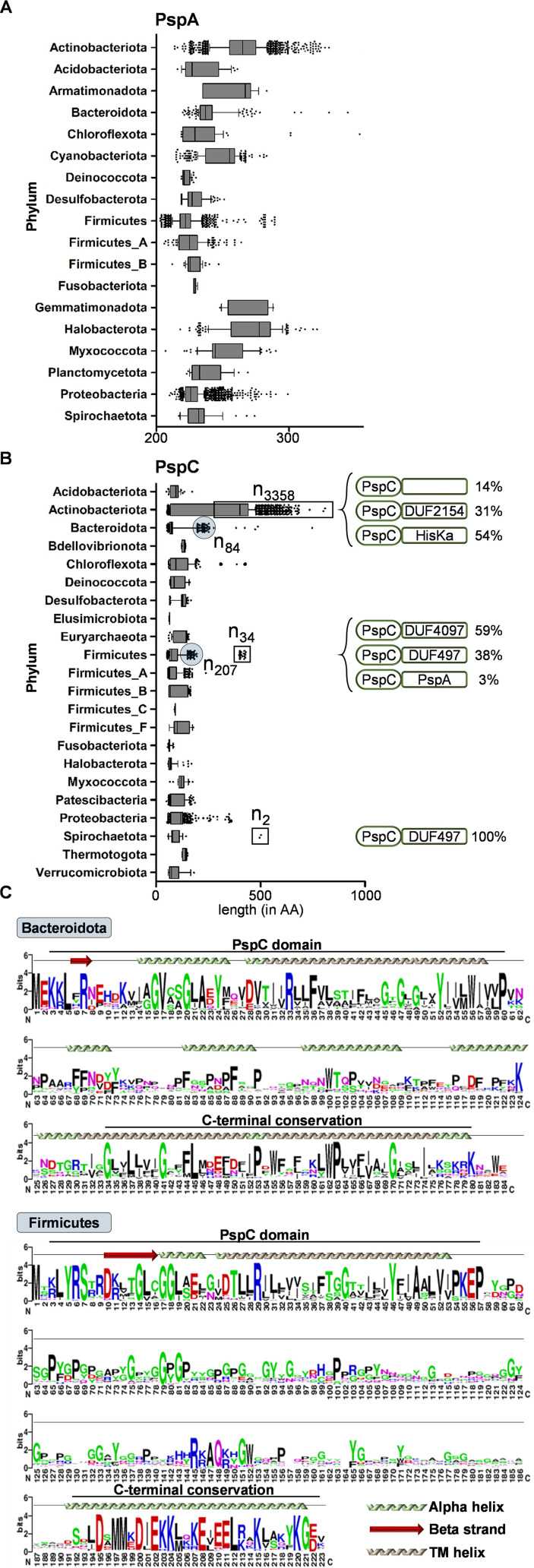
Protein length analysis of PspA and PspC. (A and B) Amino acid (AA) length distribution of PspA and -C proteins found in the depicted phyla. Phyla with more than 10 proteins were considered (see Data_S4 and Data_S5 at OpARA [http://dx.doi.org/10.25532/OPARA-117]). (C) Multiple-sequence alignment of C-terminal conserved regions of PspC proteins within the *Bacteroidota* and *Firmicutes*. Proteins highlighted in panel B were considered (see Data Sets S1 and S2 in the supplemental material; also see Data_S6 at OpARA [http://dx.doi.org/10.25532/OPARA-117]). TM, transmembrane.

We next focused our attention on the predominant PspA and PspC domains and the architectures of the cognate proteins in order to identify domain combinations that have been established and conserved in the course of evolving Psp-like responses. Such conserved domain combinations might provide important mechanistic insights into the regulation of Psp responses. For example, in C. glutamicum, a PspC domain was found to be part of a histidine kinase, indicating that PspC-dependent sensing is transduced by a two-component system in order to orchestrate a CESR in this actinobacterium (32). Toward this end, we calculated the sequence lengths of PspA and PspC domain-containing proteins. Since protein domains are on average 100 amino acids (aa) long, typically ranging from 50 to 200, we expected that the shuffling of domain architectures within one protein would result in a notable extension of protein length (42, 43). For the PspA domain-based search, we used the Pfam PspA_IM30 HMM of 221 aa in length (44) (see Materials and Methods). Analysis of the length distribution of all PspA domain-containing proteins revealed that the majority of proteins are approximately 200 to 250 aa long, indicating no significant sequence space for additional protein domains (Fig. 4A; see also Data_S6 at OpARA [http://dx.doi.org/10.25532/OPARA-117]). One notable exception was the increased size of PspA domains found in the *Actinobacteriota*: in this phylum, numerous proteins were approximately 300 aa in length. However, a subsequent analysis, using the HMMscan module, failed to identify any additional PspA-associated domains (see Data_S8 at OpARA [http://dx.doi.org/10.25532/OPARA-117]). Following this, we also predicted the secondary structure of the same subset of proteins and observed predominantly alpha helices within the C-terminal sequence space (see Data_S8 at OpARA [http://dx.doi.org/10.25532/OPARA-117]). These structural properties could point to additional functional implications, which would need further investigations.

In contrast, the analysis of PspC-containing proteins identified diverse domain architectures. We found that the majority of proteins are far longer than the ~50 aa typical for the stand-alone PspC domain (Fig. 4B) (44). In the *Actinobacteriota*, the size range of PspC-containing proteins was between 50 and 1,000 aa (Fig. 4B). A detailed analysis of all protein sequences longer than 300 aa revealed that more than 50% of them were accompanied by a histidine kinase domain (see Data_S5 at OpARA [http://dx.doi.org/10.25532/OPARA-117] for details), substantiating the diverse role of PspC in signal transduction processes within this phylum (32, 34). Conserved combinations of PspC with other domains were not restricted to *Actinobacteriota*. In *Firmicutes* and *Spirochaetota*, we observed PspC domains arranged with several domains of unknown function (DUFs). Notably, in *Firmicutes*, we identified proteins combining PspC and PspA domains, which demonstrates the existence of alternative Psp architectures compared to those found in E. coli, in which the domains are encoded by separate genes (Fig. 4B; see also Data_S5 at OpARA [http://dx.doi.org/10.25532/OPARA-117]). Additionally, we identified phylum-specific (*Bacteroidota* and *Firmicutes*) C-terminal conserved regions in PspC that did not match any protein domain model from public databases (Fig. 4C; see also Data_S6 at OpARA [http://dx.doi.org/10.25532/OPARA-117]). A previous report demonstrated that the C-terminal region of PspC is of particular importance in the secretin-dependent induction of a Psp response in Y. enterocolitica (29). Thus, we hypothesize that this conserved region found in representatives of the *Bacteroidota* and *Firmicutes* might also perform signaling functions in their respective Psp networks. To obtain a complete picture of Psp network architectures, we next expanded our analysis to the conservation of the genomic neighborhood of PspA- and PspC-encoding genes.

### Genomic context conservation of PspA- and PspC-encoding genes.

In prokaryotes, genes are often organized into operons that encode physically interacting proteins (45–48) or proteins from the same functional pathway (49, 50). The systematic association between functionally related genes in operons is frequently used to characterize genes with unknown function (51–53). The intergenic distance and orientation of individual genes are usually reliable measures to predict operon arrangements (54). It is well established that intergenic regions between identically oriented genes with less than 100- to 200-bp gaps enable accurate predictions of operon structures (55–58).

**FIG 5:**
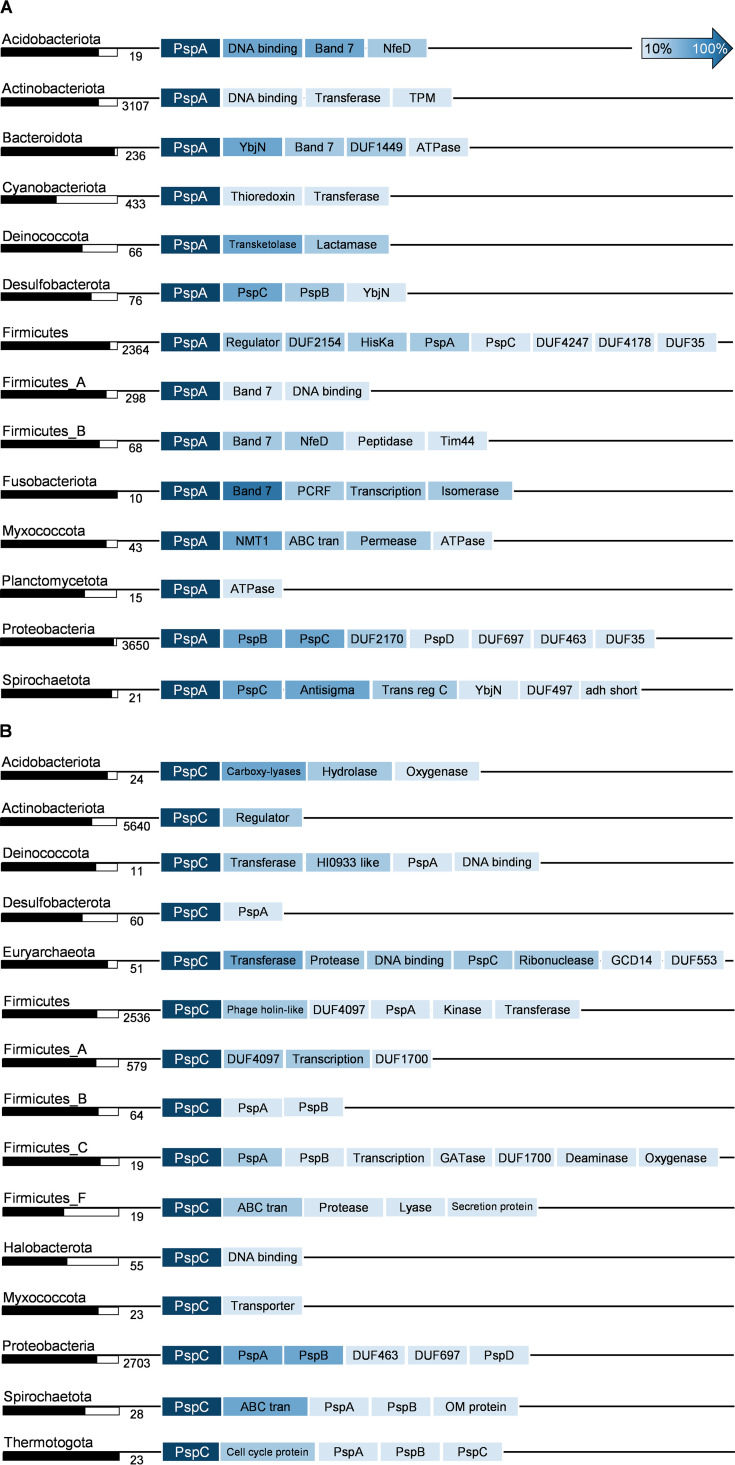
Gene neighborhood analysis of PspA and PspC. Shown are consensus gene neighborhoods for PspA and -C proteins based on domain abundance within the depicted phyla. Black/white columns indicate fractions of the respective proteins encoded in operons dependent on the total amount of proteins accounted for (the number next to the column). For details, see Materials and Methods and Data_S7 at OpARA (http://dx.doi.org/10.25532/OPARA-117).

For our analyses of PspA- and PspC-encoding gene neighborhoods, an operon was defined as genes of the same orientation that are closer than 150 bp to each other (see Materials and Methods). We generated gene neighborhood profiles for phyla containing more than 10 PspA or PspC domains (Fig. 5A and B; for full data sets, see Data_S7 at OpARA [http://dx.doi.org/10.25532/OPARA-117]). Subsequently, protein sequences of the potentially coexpressed genes were retrieved, and protein domains were identified using HMMscan (see Materials and Methods). We then created consensus gene neighborhoods based on the abundance of protein domains within each phylum (Fig. 5A and B; see also Data_S7 at OpARA [http://dx.doi.org/10.25532/OPARA-117]). As expected for *Proteobacteria*, PspA was predominantly accompanied by PspB, PspC, and PspD, reflecting the well-studied *psp* operon of E. coli. PspE was missing from the consensus gene neighborhood despite its high abundance within some orders of the *Proteobacteria* (Fig. 2B). Our analysis also reinforced previous observations that the Psp operon is often located with *ycjX*-like genes, containing the DUF463 domain restricted to the phylum *Proteobacteria* (33). DUF463 belongs to the Pfam superfamily “P-loop_NTPase (CL0023),” which contains many proteins that are involved in the assembly and function of protein complexes (37). However, a physiological link of the Psp response with these proteins is still unknown. Moreover, DUF463-containing proteins are not mandatorily associated with the Psp domain-containing proteins, as DUF463 is also found in 14% of all Psp-null genomes (7,171) (Fig. 3A), most of which again belong to the phylum *Proteobacteria* (see Data_S8 at OpARA [http://dx.doi.org/10.25532/OPARA-117]). Beyond the *Proteobacteria*, the core PspABC protein set was conserved only on genome location within the phylum *Desulfobacterota*. In most phyla, *pspA* is located without any other classical Psp domain in its neighborhood, with the exception of PspC. In a notable number of phyla, such as *Acidobacteriota*, *Bacteroidota*, *Firmicutes*, and *Fusobacteriota*, proteins containing the stomatin-like integral membrane band 7 domain were found encoded next to *pspA* genes. The presence of *pspA* genes in actinobacterial operons encoding histidine kinases suggests alternative ways of regulating PspA domain-involved and -mediated (envelope) stress responses. Our analysis demonstrated an overall tendency for *pspA* genes to be colocated with genes encoding DNA binding proteins (helix-turn-helix [HTH] domains) or other regulatory domain-containing proteins, e.g., in the phylum *Acidobacteriota*, *Firmicutes*, or *Spirochaetota* (Fig. 5A). Previously, this was observed in just a few model organisms; e.g., *pspA* is located adjacent to *pspF* in E. coli and Y. enterocolitica (33). In the phylogenetically ancient *Cyanobacteriota*, more than half of the PspA proteins are encoded as monocistronic transcriptional units, which could reflect the stand-alone characteristics of PspA as a signal preceptor and effector in a single protein (33). For PspC, the gene neighborhood analysis revealed a clear preference for genes encoding predicted membrane-associated proteins (Fig. 5B). In the majority of the analyzed phyla, PspC was encoded together with PspA and DUF proteins as well as ABC transporters and other membrane proteins. In contrast to PspA, which is preferentially encoded in operons, PspC genes are regularly located outside an operon structure (Fig. 5B, black/white bars). In phyla encoding PspC in the absence of PspA, PspC-containing proteins were predominantly accompanied by genes encoding transporters or DNA binding proteins. Especially in *Actinobacteriota* and *Halobacterota*, PspC appears to be primarily involved in cellular signaling without PspA contribution. This is in line with the observed presence of the PspC domain as a sensory unit of histidine kinases and as a part of signal transduction pathways, as discussed above (Fig. 4B).

### Psp interaction network in Bacillus subtilis.

**FIG 6:**
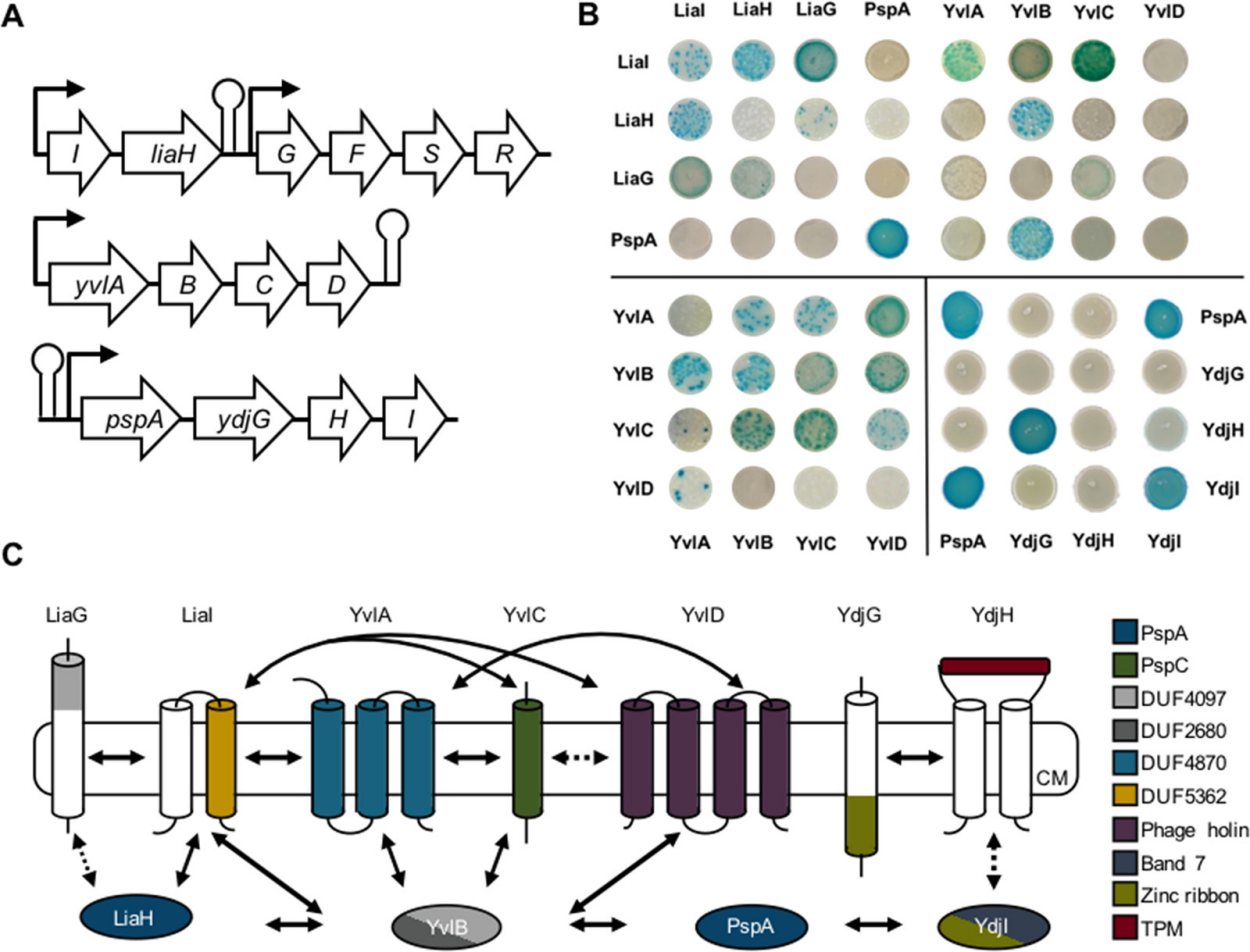
Psp network in B. subtilis. (A) Genomic organization of the Psp network across three separate operons in the B. subtilis genome. (B) Representative B2H interactions of B. subtilis Psp network proteins. (For the full data set, see Fig. S1 and S2 in the supplemental material.) (C) Schematic representation of the B2H results, comprising all members of the Psp network in B. subtilis. Protein domains are indicated by color, and protein-protein interactions are highlighted as full or, in the case of partial interactions, dotted arrows.

Our analysis of a phylogenetically diverse genomic data set revealed that PspA and PspC are the most widely distributed domains that launch a Psp response system, which indicates their ancient origin. Our study demonstrated that there is no “typical” Psp network architecture: the paradigmatic Psp responses described for E. coli and Y. enterocolitica represent just one type of Psp system, and a plethora of alternative Psp network architectures exist in the microbial world. As a first proof of principle, we analyzed the Psp domains identified in the Gram-positive model organism B. subtilis and probed if they work in concert on the protein level. The genome of B. subtilis encodes two PspA homologs (PspA and LiaH) and one PspC homolog (YvlC), which are spread across three operons. We hypothesized that most of the additional 11 genes in these operons encode proteins that partake in the Psp-centered CESR network (Fig. 6). A tight physiological and regulatory link between the proteins encoded in the *liaIH-liaGFSR* locus has been made (30, 59, 60). While the regulatory role of the LiaFSR “three”-component system in orchestrating the expression of the *liaIH* operon as the main effector of the Lia response is firmly established, no function could so far be attributed to LiaG. This membrane-anchored protein contains a DUF4097 domain, which can also be found in another hypothetical protein, YvlB, which is encoded in the *yvlABCD* operon. Remarkably, this domain has been found in the genomic neighborhood of the PspC-domain encoding protein YvlC in B. subtilis as well as in other *Firmicutes*. The remaining three genes in this operon encode putative membrane proteins. The second PspA homolog, namely, PspA, is encoded in the *pspA-ydjGHI* operon, where three genes encode additional membrane proteins and a cytoplasmic protein. One of the transmembrane proteins, YdjG, contains a zinc ribbon domain (Pfam, TF_Zn_Ribbon) that is known to bind DNA and therefore to be involved in regulatory processes within the cell. The cytoplasmic protein YdjI belongs to the band 7 protein family.

10.1128/msystems.01348-21.1 FIG S1 Overview and summary of B2H analysis. Download FIG S1, DOCX file, 0.4 MB. Copyright © 2022 Popp et al. 2022 Popp et al. https://creativecommons.org/licenses/by/4.0/ This content is distributed under the terms of the Creative Commons Attribution 4.0 International license.

10.1128/msystems.01348-21.2 FIG S2 Colony color of protein combinations within the B2H analysis. Download FIG S2, DOCX file, 0.5 MB. Copyright © 2022 Popp et al. 2022 Popp et al. https://creativecommons.org/licenses/by/4.0/ This content is distributed under the terms of the Creative Commons Attribution 4.0 International license.

For a complete picture of the protein interaction network of the Psp domains present in B. subtilis, we performed a bacterial two-hybrid (B2H) screen with all proteins from three operons encoding a Psp member (see Fig. S1 and S2 in the supplemental material). The B2H is a powerful and sensitive tool for detecting not only stable interacting proteins but also weak and transient protein interactions (61, 62). The B2H showed strong protein-protein interactions not only between proteins encoded in the same operon but also with proteins encoded in other operons (Fig. 6). The membrane anchor protein LiaI strongly interacted with the PspA homolog LiaH, which substantiates a previous study demonstrating that LiaI serves as the membrane anchor for LiaH upon cell envelope damage (30, 60). Moreover, we observed a strong LiaI and LiaG interaction and a weak interaction of LiaG with LiaH. To date, the role of LiaG in the Lia CESR remains elusive since an LiaG deletion has no observable phenotype (59). Our data indicate that LiaG might promote the recruitment of LiaH to the membrane by binding the protein directly and/or supporting the LiaI-LiaH complex (Fig. 6C). Furthermore, LiaG contains an extracellular DUF4097 domain containing a β-propeller motif. β-Propeller motifs are assumed to be involved in signal transduction and protein-protein interactions (63). The same domain occurs within the YvlB protein (Fig. 6C). Notably, YvlB strongly interacts with PspA and LiaH as well as with other proteins encoded in the *yvl* operon (Fig. 6B and C; Fig. S1 and S2). YvlB appears to play a key role in connecting the PspA homolog-encoding operons with the *yvl* operon containing the PspC homolog YvlC (Fig. 6C). Regarding the protein-protein interaction between PspA and the Ydj proteins, PspA is potentially recruited to the cell membrane and seems to interact with YdjG or YdjH via the adapter protein YdjI that contains a band 7 domain and belongs to the flotillin protein family. Flotillins are described to be involved in the organization of membrane microdomains or so-called lipid rafts and have been observed to be linked to a Psp response (64, 65). In summary, by our computational approach, we identified Psp domains spanning different loci within the genome of B. subtilis. Subsequently, and for the first time, we describe that these Psp domain-containing proteins form a network by depicting protein-protein interactions to presumably work in concert upon CES.

### Psp systems in archaea.

To date, Psp networks in archaea are largely unknown. We analyzed 915 archaeal genomes representing 10 phyla (Data Set S2). Similar to bacteria, PspA and PspC domains were both the most prevalent and taxonomically widely distributed, with 25% and 12% of the analyzed genomes containing either protein solely, respectively. In contrast, more than 50% of all genomes lacked any of the Psp domains, arguing for an overall rather low distribution of Psp members across archaea (see Data_S8 at OpARA [http://dx.doi.org/10.25532/OPARA-117]). One archaeal genome, *Methanoperedens* sp. strain BLZ2, classified as belonging to the *Halobacterota*, carried genes for two PspAs, one PspB, and one PspC domain. Strikingly, all these proteins are encoded in separate operons. The PspA domain-containing proteins are located next to genes encoding a small multidrug exporter or an extracellular peptidase. PspB and PspC are parts of operons that also contain genes encoding multiple DNA binding HTH domains and combined PAS domain-containing or response regulator proteins, indicating their involvement in signal transduction. Proteomic analysis of Haloferax volcanii revealed the upregulation of a PspA homolog upon salinity stress (66). This observation indicates a functional overlap between Psp responses in archaea and bacteria. Applying our thresholds for an operon structure (see Materials and Methods), we identified the fourth transmembrane protein encoded downstream of *pspA* (locus tag Hvo2637). Subsequent analysis identified two domains in this protein, bPH_4 and EphA2_TM, with probabilities of 60% and 80%, respectively (67). EphA2_TM has been associated with tyrosine kinase acceptors (68), which might play a signaling role within the Psp response of H. volcanii or at least serve as a membrane anchor. Psp members seem poorly conserved within archaeal representatives. Most genomes do not carry Psp-associated genes, whereas the majority of those that do carry them are limited to PspA or PspC (see Data_S8 at OpARA [http://dx.doi.org/10.25532/OPARA-117]).

10.1128/msystems.01348-21.6 DATA SET S1 Phyre2 codomain occurrence analysis of PspA or PspC domain-containing proteins in *Bacteroidota*. Download Data Set S1, PDF file, 0.07 MB. Copyright © 2022 Popp et al. 2022 Popp et al. https://creativecommons.org/licenses/by/4.0/ This content is distributed under the terms of the Creative Commons Attribution 4.0 International license.

10.1128/msystems.01348-21.7 DATA SET S2 Phyre2 codomain occurrence analysis of PspA or PspC domain-containing proteins in *Firmicutes*. Download Data Set S2, PDF file, 0.07 MB. Copyright © 2022 Popp et al. 2022 Popp et al. https://creativecommons.org/licenses/by/4.0/ This content is distributed under the terms of the Creative Commons Attribution 4.0 International license.

### Concluding remarks.

**FIG 7:**
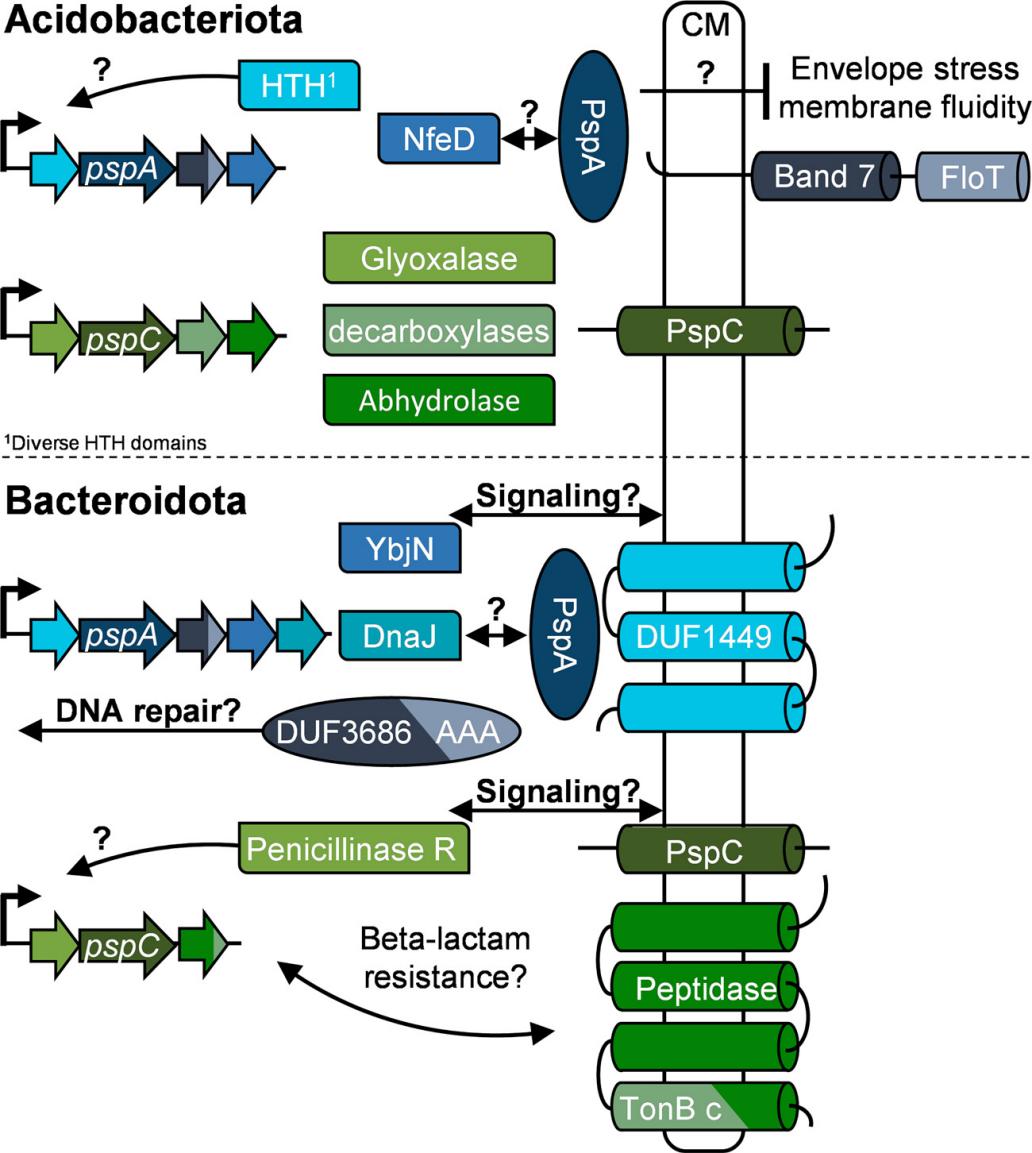
Psp network predictions. Phylum-specific *in silico* predictions of PspA and PspC networks are shown. Potential protein-protein interactions are indicated by arrows, and physiological roles are implied. Data are derived from gene neighborhood, HMMscan, and THMM analyses (for the full data set, see Data_S9 at OpARA [http://dx.doi.org/10.25532/OPARA-117]).

Previous studies on the diversity of the Psp system were limited to three bacterial phyla (33, 34, 36). We substantially expanded previous analyses by carrying out an unbiased comparative genomic analysis of Psp response networks across more than 100 phyla of bacteria and archaea. We performed Psp domain model-based searches and revealed a variety of Psp architectures and a heterogeneous distribution of Psps across taxonomic groups. We demonstrated that PspA and PspC domains are both the most frequently found and phylogenetically most widely distributed components of the Psp system (Fig. 2A). Our analysis revealed diverse protein networks of Psp responses across bacteria and archaea. The Psp system displays remarkable diversity with respect to component design, phylum-specific conserved architectures, and postulated modes of both signal transduction and underlying physiologies. We fully reconstructed *in silico* Psp networks in two phyla that contain large numbers of sequenced genomes: *Acidobacteriota* and *Bacteroidota*. For example, in *Acidobacteriota*, we found PspA associated with band 7 and FloT domain-containing proteins responsible for stabilizing membrane integrity upon changes in the fluidity state under stress conditions (69). The NfeD protein, which has been identified as a partner protein in gene neighborhood analyses, may support PspA assembly and/or function (70) (Fig. 7). We also identified the HTH domain-containing transcriptional regulators encoded in the PspA operons that potentially regulate operon expression. Our analysis suggests that PspC in *Bacteroidota* might be directly involved in the beta-lactam stress response (Fig. 7).

We demonstrated the utility of our computational analysis by showing that proteins containing Psp domains in B. subtilis physically interact, although they are encoded in three different operons (Fig. 6; Fig. S1 and S2). It is well known that the Lia system containing the PspA homolog LiaH serves as a resistance determinant under CES conditions (71–73). The interaction of LiaH with the YvlB protein revealed in this study suggests a potential additional role for LiaH bridging the Lia system to other Psp systems in B. subtilis. Domain reoccurrence was further observed for the negative regulator of the LiaRS TCS, LiaF. This protein contains a DUF2154 domain that we also identified in *Actinobacteria* to be fused with PspC (Fig. 4B).

Furthermore, it is tempting to speculate that YvlB, which interacts with individual proteins of all Psp domain-containing operons, binds PspA and/or LiaH and subsequently interacts with one of the Yvl membrane-anchored proteins to support cell envelope integrity at the site of damage perception. However, the detailed binding events of the network in temporal and spatial occurrence cannot be extracted by the B2H results. Nevertheless, this implies that the PspA homologs are not only acting within their distinct and assumed function together with their own operon partner proteins but also functionally cross-linked to proteins of other loci, here the *yvl* locus (Fig. 6). However, details on the mechanistic and functional role of YvlB in the Psp response in B. subtilis are still unknown and under investigation in our laboratory. Interestingly, the majority of membrane-directed proteins of the Psp-spanning network in B. subtilis showed strong interactions despite their genomic organization and regulation. Diverse systems have been shown to be induced by specific membrane-interfering conditions but also work in concert and orchestrate a widespread CESR when needed (5). Thus, the interaction of the membrane proteins within these modules corroborates the cross talk and redundancy of the CESR in B. subtilis (Fig. 6C).

In summary, our comprehensive and unbiased analysis of bacterial and archaeal genomes revealed an unexpected diversity of Psp systems that can now be tested experimentally. We believe that reoccurring patterns of Psp domains and the well-known concept of domain shuffling within organisms can give rise to protein networks that work in concert, thus spanning a whole new perception of Psp domain-centered (envelope) stress responses in the microbial landscape.

## MATERIALS AND METHODS

### Bioinformatics tools and software environments.

The following software packages were applied in this study: HMMER v3.2.1 (74), the MAFFT 7 online version (75), MEGA-X (76), CDvist (67), Jalview (77), iTOL (78), and CD-HIT (79). Multiple-sequence alignments (MSAs) were generated using the default-set L-IN-I algorithm from MAFFT (75). The neighbor-joining tree (Fig. 2B; see also Data_S2 at OpARA [http://dx.doi.org/10.25532/OPARA-117]) was built in MEGA-X with pairwise deletion using the Poisson model. All maximum likelihood trees were computed in MEGA-X by applying the Jones-Taylor-Thornton (JTT) substitution model and using all sites unless otherwise specified. Final visualization and mapping of Psp domains onto the phylogenetic trees were performed using iTOL. Computational analyses were executed on a local computing environment, and custom scripts for data processing, filtering, and evaluation were written in Python v2.7 or v3.7 in the environment of PyCharm v2019.1.1.

### Data sources.

Bacterial and archaeal genomes were analyzed that are declared to be representatives according to the Genome Taxonomy Database (GTDB) r86 (35). In total, 22,254 proteomes were obtained that were available at the National Center for Biotechnology Information (NCBI) Reference Sequence (RefSeq) or GenBank database as of October 2018 (see Data_S1 at OpARA [http://dx.doi.org/10.25532/OPARA-117]). All analyses were done using this final data set or derived subsets as indicated. Analysis of Psp domains in the obtained genomes was performed using HMMer (http://hmmer.org/). To run the HMMsearch module, hidden Markov models (HMMs) were obtained from the Protein Families (Pfam) database: PF04012.12, PF06667.12, PF04024.12, PF09584.10, and PF09583.10 (44). Models for the two remaining Psp-related domains, PspE and PspF, for which no model was available at Pfam, were generated by downloading their TIGRFAMs, TIGR02981 and TIGR02974, respectively (80). The seed sequences were obtained and used to build HMMs locally using the HMMbuild module.

### Identification of Psp domains in the genomic data sets.

All seven, PspA to PspG, HMMs were probed against each genome downloaded by applying the HMMsearch module. The obtained results were checked for their accuracy and to avoid false-positive hits via a Python script that filtered according to the following criteria: (i) the E value threshold cutoff for a positive hit was set to 10^−3^, (ii) the domain had to start within the first 50 aa of the protein, and (iii) the alignment of the HMM with the protein sequence had to exceed 90% of the model length. In total, 39,950 proteins fulfilled these criteria and were considered Psps (see Data_S1 at OpARA [http://dx.doi.org/10.25532/OPARA-117]).

### Construction of phylogenetic trees.

The sets of the concatenated and aligned 120 proteins (bacterial) and 122 proteins (archaeal), proposed by the GTDB criteria, were used to build the phylogenetic trees in Fig. 2 (35). Figure 2A was adapted and modified from the precomputed phylogenetic tree presented by AnnoTree (81) and serves only as a representation of the phylogenetic distribution of Psps. For the data set in Fig. 2B, a maximum of 55 members of each of the 53 orders within the class *Gammaproteobacteria* were randomly chosen, resulting in 742 genomes (see Data_S2 at OpARA [http://dx.doi.org/10.25532/OPARA-117]). An amount of 55 genomes was set according to a hypergeometric distribution, setting a 95% probability threshold to include at least one genome within the *Enterobacterales* order having the full set of Psps. From orders with fewer than 55 members, all genomes were included, and a neighbor-joining tree was computed. The data set in Fig. 2C is based on the species (41 genomes) used previously (82), representing the diversity of the *Enterobacteriaceae* family (see Data_S2 at OpARA [http://dx.doi.org/10.25532/OPARA-117]). Four species had to be replaced by genomes declared to be representatives according to the GTDB. A maximum likelihood tree using the JTT model with 100 bootstraps was applied to the respective sets of 120 concatenated aligned proteins provided by the GTDB (35).

### Protein domain cooccurrence analysis.

To characterize potential cooccurring protein domains associated with PspA or PspC domain-containing proteins, the respective proteins were downloaded from the NCBI protein database (see Data_S4 and Data_S5 at OpARA [http://dx.doi.org/10.25532/OPARA-117]). The HMMscan module was used with an E value threshold of 0.001 to search for associated domains in PspA and PspC domain-containing proteins. The identified domains were obtained from the Pfam HMM (March 2019) library for Pfam-A families. For a better overview, only phyla containing more than 10 Psps were included in Fig. 3A and B. For the full data set, see Data_S4 and Data_S5 at OpARA (http://dx.doi.org/10.25532/OPARA-117).

### Creation of WebLogos for PspC domain-containing proteins.

To identify PspC C-terminal conserved regions, the respective proteins were further analyzed by generating an MSA in MAFFT (75) and using Jalview for visualization (77) (Fig. 4C). The WebLogo of the final MSA was created using the online version of WebLogo (83). Secondary structure predictions were performed in Phyre2 (84). For the proteins used, see the MSA provided in Data Sets S1 and S2 in the supplemental material (data not shown).

### Gene neighborhood analysis.

Analysis of gene neighborhoods was performed using the application programming interface (API) implemented in the Microbial Signal Transduction database (MiST3) (85). For each query gene, five up- and downstream genes were obtained and filtered for their orientations and their respective locations. An operon structure was defined for genes that shared the same strand orientation and were located no more than 150 bp apart from each other. Proteins that were identified as belonging to a gene neighborhood were then obtained from the RefSeq NCBI database. The HMMscan module was then used to identify protein domains, using an E value threshold of 0.001. To exclude overlapping domain hits, e.g., band 7 and band 7_1, which would cover the same sequence space, only the first domain hits were considered. For proteins containing multiple nonoverlapping domains, their domain architecture was fused, e.g., HisKA_3 and HATPase_c, and finally categorized (Suppl. Data_S9 and S10 at OpARA [http://dx.doi.org/10.25532/OPARA-117]). For a better overview and to generate the consensus gene neighborhood, two thresholds were applied: (i) only phyla containing at least 10 Psp-positive genomes were included, and (ii) the identified protein domain had to be present in more than 10% of the genomes within the analyzed phyla; e.g., of the 19 PspAs analyzed in *Acidobacteriota*, 11 contained a band 7_Flot protein in their gene neighborhood, thus resulting in 58%. For the complete data set, see Data_S9 at the URL mentioned above.

### DNA manipulation.

Plasmids were constructed using standard cloning techniques as described previously (86). For DNA amplification via PCR, Q5 polymerase was used. Enzymes were purchased from New England BioLabs (NEB) (Ipswich, MA, USA) and applied according to their respective protocols. Positive E. coli clones were checked by colony PCR using OneTaq polymerase. All constructs were verified by sequencing. All strains, primers, and plasmids used in this study are listed in Tables S1 to S3 in the supplemental material.

10.1128/msystems.01348-21.3 TABLE S1 Strains used in this study. Download Table S1, DOCX file, 0.01 MB. Copyright © 2022 Popp et al. 2022 Popp et al. https://creativecommons.org/licenses/by/4.0/ This content is distributed under the terms of the Creative Commons Attribution 4.0 International license.

### Bacterial two-hybrid assay.

The bacterial two-hybrid experiment is based on an adenylate cyclase reconstruction resulting in the transcription of the reporter gene *lacZ* in E. coli (87). The gene encodes a β-galactosidase. The enzyme is able to cleave 5-bromo-4-chloro-3-indolyl-β-d-galactopyranoside (X-Gal), which results in blue colonies. For the bacterial two-hybrid assay, the adenylate cyclase is divided into two parts, each of them either N- or C-terminally present on the vector pUT18/pUT18C or pKT25/pKT25N. Genes of interest, encoding candidates for protein-protein interaction, were cloned into these vectors and cotransformed into E. coli BTH101. Because the interaction of the proteins can be influenced by the position of the adenylate cyclase, all plasmid combinations were used. The vectors pUT18 zip and pKT25N zip were included and served as positive controls, and the empty vectors pUT18 and pKT25 were used as negative controls. After transformation, the cells were pelleted and resuspended in 40 μL LB medium. Ten microliters of each transformation mix was spotted onto agar plates containing ampicillin (100 μg mL^−1^), kanamycin (50 μg mL^−1^), isopropyl-β-d-thiogalactopyranoside (IPTG) (0.5 mM), and X-Gal (40 μg mL^−1^). After the spots dried, the procedure was repeated. The plates were incubated at 30°C overnight, and the next day, the rest of the transformation mix was spotted two times. In most cases, not enough colonies grew to cover the whole spot area. To achieve a higher colony density, cultures of the different strains grown overnight were prepared, and 2 10-μL aliquots were spotted the following day. The plates were wrapped in aluminum foil to protect them from incident light exposure and stored in a fridge for several weeks to increase the contrast and intensity of the colony color.

### Data availability.

The data underlying this article are available in the article, in its online supplemental material, on OpARA (http://dx.doi.org/10.25532/OPARA-117), and upon request to the corresponding authors.

10.1128/msystems.01348-21.4 TABLE S2 Plasmids used in this study. Download Table S2, DOCX file, 0.01 MB. Copyright © 2022 Popp et al. 2022 Popp et al. https://creativecommons.org/licenses/by/4.0/ This content is distributed under the terms of the Creative Commons Attribution 4.0 International license.

10.1128/msystems.01348-21.5 TABLE S3 Oligonucleotides used in this study. Download Table S3, DOCX file, 0.01 MB. Copyright © 2022 Popp et al. 2022 Popp et al. https://creativecommons.org/licenses/by/4.0/ This content is distributed under the terms of the Creative Commons Attribution 4.0 International license.
