# Supplementary material for: Phyletic Distribution and Diversification of the Phage Shock Protein Stress Response System in Bacteria and Archaea
Source: mSystems. 2022 May 23;7(3):e01348-21. doi: 10.1128/msystems.01348-21 (PMC9239133; doi:10.1128/msystems.01348-21)
Supplement: FIG S1 [file msystems.01348-21-s0001.docx]

Figure S1

Overview and Summary of protein-protein interactions within the PSP network in *Bacillus subtilis* determined by B2H assays. Protein encoding genes of the Lia-system regulated by the LiaRS two-component system are marked in blue. Protein expression of loci containing the *yvl*- or *pspA*-*ydj* genes that are the control of the extracytoplasmic function factor σ^W^, highlighted in green. Dark blue filled circles indicate strong protein-protein interaction whereas circles in light blue point weak interactions. White circles stand for no protein-protein interaction. For experimental details please see the Material and Methods section.
