# Supplementary material for: Phyletic Distribution and Diversification of the Phage Shock Protein Stress Response System in Bacteria and Archaea
Source: mSystems. 2022 May 23;7(3):e01348-21. doi: 10.1128/msystems.01348-21 (PMC9239133; doi:10.1128/msystems.01348-21)
Supplement: FIG S2 [file msystems.01348-21-s0002.docx]

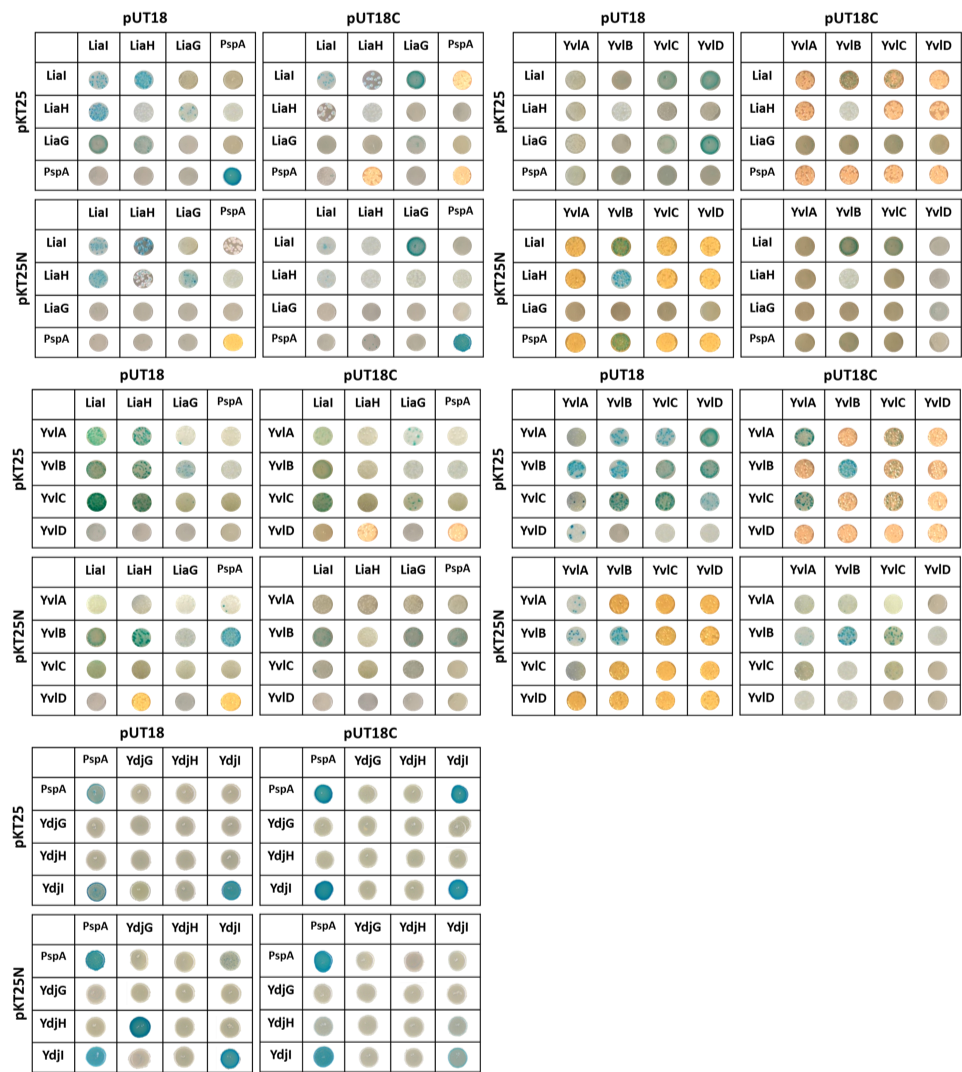


Figure S2

Colony colour of protein combinations within the PSP response in *Bacillus subtilis* tested by B2H assay. Please see Material and Methods for experimental details.
