# Supplementary material for: Phyletic Distribution and Diversification of the Phage Shock Protein Stress Response System in Bacteria and Archaea
Source: mSystems. 2022 May 23;7(3):e01348-21. doi: 10.1128/msystems.01348-21 (PMC9239133; doi:10.1128/msystems.01348-21)
Supplement: DATA SET S1 [file msystems.01348-21-s0003.pdf]

Phyre2

Email	popp.philipp@gmail.com
Description	Weblogo_Bacteriodota
Date	Wed Sep 11 15:40:48 BST 2019
Unique Job ID	7cf38cdd617c6372

Secondary structure and disorder prediction

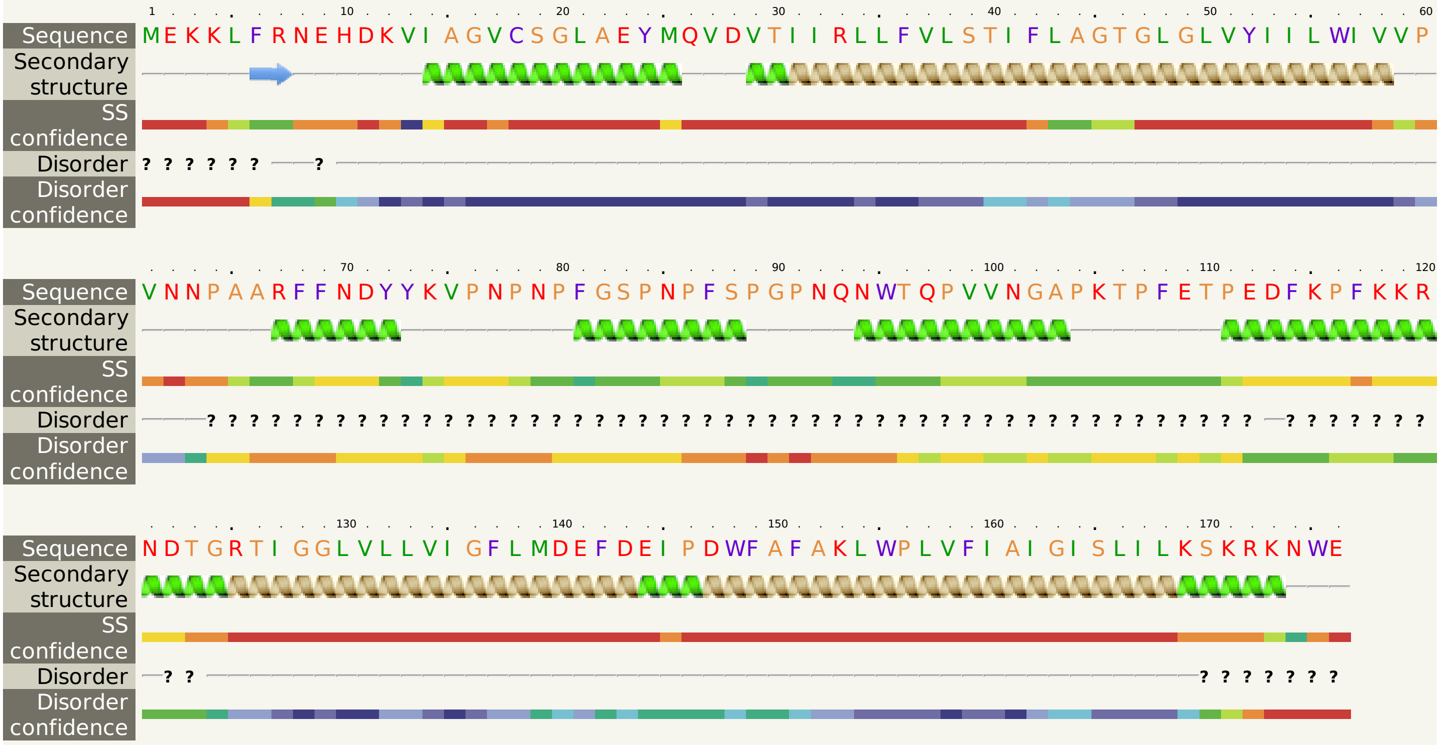

Confidence Key

High(9) Low (0)

? Disordered (41%)

Alpha helix (73%)

Beta strand (1%)

TM helix (39%)
