# Supplementary material for: Phyletic Distribution and Diversification of the Phage Shock Protein Stress Response System in Bacteria and Archaea
Source: mSystems. 2022 May 23;7(3):e01348-21. doi: 10.1128/msystems.01348-21 (PMC9239133; doi:10.1128/msystems.01348-21)
Supplement: DATA SET S2 [file msystems.01348-21-s0004.pdf]

Email	popp.philipp@gmail.com
Description	Weblogo_FIRMICUTES
Date	Wed Sep 11 15:44:30 BST 2019
Unique Job ID	a1a798ec058c7191

Secondary structure and disorder prediction

	1–60
Sequence	MTKLYRSTRDKLTGLCGGLAELGIDTLLRILLVVSIFTGGTTILIYFIAALVIPKEPYGP
Disorder	? ? ? ? ? ? ? … ? ? ? ? ?

	61–120
Sequence	DSGPYGPGPGAPYGPYGGPGPYYGPGPGGYYGYDHGPPRGPYNNGGYGDPGGGGYGPPGG
Disorder	? ?

	121–180
Sequence	GAYGGRPPKHHRAQKHGWGPPQPGGYYYGPYGAGGAAADSDLDSMMKDIEKKLQKEIEE
Disorder	? ?

	181–192
Sequence	LRAKLAKYKGEV
Disorder	… ? ? ? ? ?

Confidence Key

High(9) … Low (0)

? Disordered (60%)

Alpha helix (34%)

Beta strand (3%)

TM helix (15%)
