# Supplementary material for: Phyletic Distribution and Diversification of the Phage Shock Protein Stress Response System in Bacteria and Archaea
Source: mSystems. 2022 May 23;7(3):e01348-21. doi: 10.1128/msystems.01348-21 (PMC9239133; doi:10.1128/msystems.01348-21)
Supplement: TABLE S1 [file msystems.01348-21-s0005.docx]

Table S1. Strains used in this study

| **Strain** | **Genotype *^a^*** | **Source** |
| --- | --- | --- |
| ***Escherichia coli*** | | |
| BTH 101 | F^-^ *cya*-99 *araD*139 *galE*15 *galK*16 *rpsL*1 (*str*^R^) *hsdR*2 *mcrA*1 *mcrB*1 | Lab stock |
| DH10β | F^–^ *mcr*A Δ(*mrr*-*hsd*RMS-*mcr*BC) φ80*lac*ZΔM15 Δ*lac*X74 *rec*A1 *end*A1 *ara*D139 Δ(*ara-leu*)7697 *gal*U *gal*K λ^–^ *rps*L(*str*^R^) *nup*G | Lab stock |
| XL1 blue | *rec*A1 *end*A1 *gyr*A96 *thi*-1 *hsd*R17 *sup*E44 *rel*A1 lac [F ́ *pro*AB *lac*I^q^ Z∆M15 Tn10 (*tet*^R^) | Lab stock |
| TME183 | XL1 blue pUT18-*liaI* | This study |
| TME184 | XL1 blue pUT18C-*liaI* | This study |
| TME185 | XL1 blue pKT25-*liaI* | This study |
| TME186 | XL1 blue pKT25N-*liaI* | This study |
| TME203 | XL1 blue pUT18-*liaG* | This study |
| TME204 | XL1 blue pUT18C-*liaG* | This study |
| TME205 | XL1 blue pKT25-*liaG* | This study |
| TME206 | XL1 blue pKT25N-*liaG* | This study |
| TME207 | XL1 blue pUT18-*liaH* | This study |
| TME208 | XL1 blue pUT18C-*liaH* | This study |
| TME209 | XL1 blue pKT25-*liaH* | This study |
| TME210 | XL1 blue pKT25N-*liaH* | This study |
| TME343 | XL1 blue pUT18-*pspA* | This study |
| TME344 | XL1 blue pUT18C-*pspA* | This study |
| TME345 | XL1 blue pKT25-*pspA* | This study |
| TME346 | XL1 blue pKT25N-*pspA* | This study |
| TME554 | XL1 blue pKT25N-*yvlA* | This study |
| TME555 | XL1 blue pKT25-*yvlA* | This study |
| TME556 | XL1 blue pKT25N-*yvlB* | This study |
| TME557 | XL1 blue pKT25-*yvlB* | This study |
| TME558 | XL1 blue pKT25N-*yvlC* | This study |
| TME559 | XL1 blue pKT25-*yvlC* | This study |
| TME560 | XL1 blue pKT25N-*yvlD* | This study |
| TME561 | XL1 blue pKT25-*yvlD* | This study |
| TME566 | XL1 blue pUT18-*yvlA* | This study |
| TME567 | XL1 blue pUT18C-*yvlA* | This study |
| TME568 | XL1 blue pUT18-*yvlB* | This study |
| TME569 | XL1 blue pUT18C-*yvlB* | This study |
| TME570 | XL1 blue pUT18-*yvlC* | This study |
| TME571 | XL1 blue pUT18C-*yvlC* | This study |
| TME572 | XL1 blue pUT18-*yvlD* | This study |
| TME573 | XL1 blue pUT18C-*yvlD* | This study |
| TME2876 | DH10β pUT18C-*ydjG* | This study |
| TME2877 | DH10β pUT18C-*ydjH* | This study |
| TME2878 | DH10β pUT18C-*ydjI* | This study |
| TME2880 | DH10β pUT18-*ydjG* | This study |
| TME2881 | DH10β pUT18-*ydjH* | This study |
| TME2882 | DH10β pUT18-*ydjI* | This study |
| TME2884 | DH10β pKT25-*ydjG* | This study |
| TME2885 | DH10β pKT25-*ydjH* | This study |
| TME2886 | DH10β pKT25-*ydjI* | This study |
| TME2887 | DH10β pKT25N-*ydjG* | This study |
| TME2888 | DH10β pKT25N-*ydjH* | This study |
| TME2889 | DH10β pKT25N-*ydjI* | This study |

*^a^* str streptomycin; tet tetracycline
