# Supplementary material for: Phyletic Distribution and Diversification of the Phage Shock Protein Stress Response System in Bacteria and Archaea
Source: mSystems. 2022 May 23;7(3):e01348-21. doi: 10.1128/msystems.01348-21 (PMC9239133; doi:10.1128/msystems.01348-21)
Supplement: TABLE S2 [file msystems.01348-21-s0006.docx]

Table S2. Plasmids used in this study

| **Name/Number** | **Genotype *^a^*** | **Primer** | **Source** |
| --- | --- | --- | --- |
| pUT18 | P*lac*, MCS, T18 (AA 225 to 399 of ccaA), *bla*, Ori ColE1 | -- | Karimova et al., 1998 |
| pUT18C | P*lac*, T18 (AA 225 to 399 of  ccaA), MCS, *bla*, Ori ColE1 | -- | Karimova et al., 1998 |
| pKT25 | P*lac*, T25 (first 224 AA of  ccaA), MCS, *kan*, Ori p15A | -- | Karimova et al., 1998 |
| pKT25N | P*lac*, MCS T25 (first 224 AA of ccaA) *kan*, Ori p15A | -- | Karimova et al., 1998 |
| pUT18 zip | P*lac*, T18 (AA 225 to 399 of ccaA)-leucine zipper of GCN4, MCS, *bla*, Ori ColE1 | -- | Karimova et al., 1998 |
| pUT25 zip | P*lac*, T25 (first 224 AA of ccaA)-leucine zipper of GCN4, MCS, *kan*, Ori p15A | -- | Karimova et al., 1998 |
| 248 | pUT18-*liaI* | -- | R. Emmins, Newcastle |
| 249 | pUT18C-*liaI* | -- | R. Emmins, Newcastle |
| 250 | pKT25-*liaI* | -- | R. Emmins, Newcastle |
| 251 | pKT25N-*liaI* | -- | R. Emmins, Newcastle |
| 272 | pUT18-*liaG* | -- | R. Emmins, Newcastle |
| 273 | pUT18C-*liaG* | -- | R. Emmins, Newcastle |
| 274 | pKT25-*liaG* | -- | R. Emmins, Newcastle |
| 275 | pKT25N-*liaG* | -- | R. Emmins, Newcastle |
| 310 | pKT25N-*liaH* | TM12337TM1234 | This study |
| 311 | pKT25-*liaH* | TM12337TM1234 | This study |
| 312 | pUT18C-*liaH* | TM12337TM1234 | This study |
| 313 | pUT18-*liaH* | TM12337TM1234 | This study |
| 543 | pUT18-*yvlA* | TM1924/TM1925 | This study |
| 544 | pUT18C-*yvlA* | TM1924/TM1925 | This study |
| 545 | pKT25N-*yvlA* | TM1924/TM1925 | This study |
| 546 | pKT25-*yvlA* | TM1924/TM1925 | This study |
| 547 | pUT18-*yvlB* | TM1926/TM1927 | This study |
| 548 | pUT18C-*yvlB* | TM1926/TM1927 | This study |
| 549 | pKT25-*yvlB* | TM1926/TM1927 | This study |
| 550 | pKT25N-*yvlB* | TM1926/TM1927 | This study |
| 551 | pUT18-*yvlC* | TM1928/TM1929 | This study |
| 552 | pUT18C-*yvlC* | TM1928/TM1929 | This study |
| 553 | pKT25-*yvlC* | TM1928/TM1929 | This study |
| 554 | pKT25N-*yvlC* | TM1928/TM1929 | This study |
| 555 | pUT18-*yvlD* | TM1930/TM1931 | This study |
| 556 | pUT18C-*yvlD* | TM1930/TM1931 | This study |
| 557 | pKT25-*yvlD* | TM1930/TM1931 | This study |
| 558 | pKT25N-*yvlD* | TM1930/TM1931 | This study |
| 769 | pUT18-*pspA* | TM1323/TM1324 | This study |
| 770 | pUT18C-*pspA* | TM1323/TM1324 | This study |
| 771 | pKT25-*pspA* | TM1323/TM1324 | This study |
| 772 | pKT25N-*pspA* | TM1323/TM1324 | This study |
| 2485 | pUT18C-*ydjH* | -- | M. Bramkamp, Kiel |
| 2486 | pUT18C-*ydjI* | -- | M. Bramkamp, Kiel |
| 2488 | pUT18-*ydjG* | -- | M. Bramkamp, Kiel |
| 2489 | pUT18-*ydjH* | -- | M. Bramkamp, Kiel |
| 2490 | pUT18-*ydjI* | -- | M. Bramkamp, Kiel |
| 2492 | pKT25-*ydjG* | -- | M. Bramkamp, Kiel |
| 2493 | pKT25-*ydjH* | -- | M. Bramkamp, Kiel |
| 2494 | pKT25-*ydjI* | -- | M. Bramkamp, Kiel |
| 2496 | pKT25N-*ydjG* | -- | M. Bramkamp, Kiel |
| 2497 | pKT25N-*ydjH* | -- | M. Bramkamp, Kiel |
| 2498 | pKT25N-*ydjI* | -- | M. Bramkamp, Kiel |
