# Supplementary material for: Phyletic Distribution and Diversification of the Phage Shock Protein Stress Response System in Bacteria and Archaea
Source: mSystems. 2022 May 23;7(3):e01348-21. doi: 10.1128/msystems.01348-21 (PMC9239133; doi:10.1128/msystems.01348-21)
Supplement: TABLE S3 [file msystems.01348-21-s0007.docx]

Table S3. Oligonucleotides used in this study

| **Name** | **Sequence*^a^*** |
| --- | --- |
| **Bacterial Two Hybrid Cloning** | |
| TM1233 (*liaH* fwd (*Xba*I)) | AGCG**TCTAGA**GATGGTATTAAAAAGAATCAG |
| TM1234 (*liaH* rev (*Bam*HI)) | AGCT**GGATCC**AGTTCATTTGCCGCTTTTGTCTGGTC |
| TM1323 (*pspA* fwd (*Xba*I)) | AGCT**TCTAGA**GATGAGTATAATTGGAAG |
| TM1324 (*pspA* rev (*Bam*HI)) | AGCT**GGATCC**CTCTTATCGAGCATCATTTTCGC |
| TM1924 (*yvlA* fwd (*Xba*I)) | AGCT**TCTAGA**CTTGAACCGTAATCAAGC |
| TM1925 (*yvlA* rev (*Bam*HI)) | AGCT**GGATCC**GATGCTGCACGCAGGACCTTAATG |
| TM1926 (*yvlB* fwd (*Xba*I)) | AGCT**TCTAGA**CATGAAGCAAGAAAAGGAACGAATCC |
| TM1927 (*yvlB* rev (*Bam*HI)) | AGCT**GGATCC**AACCTCTGTGAGTACTTTAG |
| TM1928 (*yvlC* fwd (*Xba*I)) | AGCT**TCTAGA**CATGAATAAGCTTTATCGCTCAG |
| TM1929 (*yvlC* rev (*Bam*HI)) | AGCT**GGATCC**AGTTTCATATCCCTTTCTGACGG |
| TM1930 (*yvlD* fwd (*Xba*I)) | AGCT**TCTAGA**CATGGTAAAATGGGCAGTCAGC |
| TM1931 (*yvlD* rev (*Bam*HI)) | AGCT**GGATCC**GGTTTTTTTCTAAGCGGCTCTAAAATGCC |
| **Bacterial Two Hybrid Cloning Check** | |
| TM1220 (pUT18 fwd) | AGCTCACTCATTAGGCACCC |
| TM1221 (pUT18 rev) | CCGTCGTAGCGGAACTGGCG |
| TM1222 (pUT18C fwd) | TCGACGATGGGCTGGGAGCC |
| TM1223 (pUT18C rev) | AGCAGACAAGCCCGTCAGGG |
| TM1224 (pKT25 fwd) | GGCGGATATCGACATGTTCG |
| TM1225 (pKT25 rev) | ATCGGTGCGGGCCTCTTCGC |
| TM1226 (pKT25N fwd) | GCTCACTCATTAGGCACCCC |
| TM1227 (pKT25N rev) | GGCGGAACATCAATGTGGCG |

*^a^* **Bold** = restriction enzyme recognition sites
